# Acetylation/deacetylation and microtubule associated proteins influence flagellar axonemal stability and sperm motility

**DOI:** 10.1042/BSR20202442

**Published:** 2020-12-02

**Authors:** Veena Chawan, Smita Yevate, Rahul Gajbhiye, Vijay Kulkarni, Priyanka Parte

**Affiliations:** 1 Department of Gamete Immunobiology, ICMR-National Institute for Research in Reproductive Health, Mumbai, India; 2 Department of Reproductive Endocrinology and Infertility, ICMR-National Institute for Research in Reproductive Health, Mumbai, India

**Keywords:** acetylation/deacetylation, HDAC6, Microtubule associated protein, Microtubule stability, Spermatozoa

## Abstract

PTMs and microtubule-associated proteins (MAPs) are known to regulate microtubule dynamicity in somatic cells. Reported literature on modulation of α-tubulin acetyl transferase (αTAT1) and histone deacetylase 6 (HDAC6) in animal models and cell lines illustrate disparity in correlating tubulin acetylation status with stability of MT. Our earlier studies showed reduced acetyl tubulin in sperm of asthenozoospermic individuals. Our studies on rat sperm showed that on inhibition of HDAC6 activity, although tubulin acetylation increased, sperm motility was reduced. Studies were therefore undertaken to investigate the influence of tubulin acetylation/deacetylation on MT dynamicity in sperm flagella using rat and human sperm. Our data on rat sperm revealed that HDAC6 specific inhibitor Tubastatin A (T) inhibited sperm motility and neutralized the depolymerizing and motility debilitating effect of Nocodazole. The effect on polymerization was further confirmed *in vitro* using pure MT and recHDAC6. Also polymerized axoneme was less in sperm of asthenozoosperm compared to normozoosperm. Deacetylase activity was reduced in sperm lysates and axonemes exposed to T and N+T but not in axonemes of sperm treated similarly suggesting that HDAC6 is associated with sperm axonemes or MT. Deacetylase activity was less in asthenozoosperm. Intriguingly, the expression of MDP3 physiologically known to bind to HDAC6 and inhibit its deacetylase activity remained unchanged. However, expression of acetyl α-tubulin, HDAC6 and microtubule stabilizing protein SAXO1 was less in asthenozoosperm. These observations suggest that MAPs and threshold levels of MT acetylation/deacetylation are important for MT dynamicity in sperm and may play a role in regulating sperm motility.

## Introduction

Spermatozoa (Sperm) flagella plays an indispensable role in procreation with its ability to steer the sperm towards oocyte thereby facilitating its fertilization. The axoneme, the crux of sperm flagellum, is surrounded by outer dense fibres (ODFs). In the midpiece, these ODFs are wrapped by an array of mitochondrial sheath, in principal piece with fibrous sheath (FS) and endpiece consist of only the axoneme. MTs comprise alternate subunits of α- and β-tubulin and give rise to the cylindrical structure called protofilaments. *In vivo*, this hollow cylindrical structure is decorated with β-tubulin demarcating positive end or growing end while the negative end is marked by γ-tubulin capped in stable structures such as centrioles and basal bodies. The property of MT protofilament to undergo growing and shrinking at their plus end is called as dynamic instability [1]. Microtubule-associated proteins (MAPs) and PTMs are known to regulate MT dynamics, and in somatic cells, they aid to perform various cellular processes such as intracellular transport, cell cycle and motility [2]. Cilia and Flagella on the contrary consist of stable axonemal MTs arranged in 9+2 pattern with 9 outer MT doublets surrounding a central pair of singlet MTs. Most of the ciliated and flagellar organisms such as *Chlamydomonas reinhardtii* (biflagellate algae) and ciliated parasite *Tetrahymena thermophillia* undergo assembly and disassembly in accordance with cell cycle and to timely facilitate the ciliary response for signalling, movement and mechanosensation [3–5].

The motility of cilia and flagella requires maintenance of cilium length through axoneme extension that is entirely governed by complex process called as intraflagellar transport (IFT) with notable exception in the mature sperm flagella [6–10]**.** The flagella of mature mammalian sperm differ from most of the ciliated and flagellated organisms in that they do not carry pool of precursors required for flagellar turnover and consist of highly stable protofilaments [11–13]**.**

MAPs and PTMs harbor on the stable axonemal protofilaments. Acetylation and detyrosination are majorly correlated with stability of MT. Acetylation occurs in luminal region of MTs at Lys 40 (K40) of α-tubulin whereas detyrosination on the C-terminal Y residue of α-tubulin. Several reports revealed that detyrosination on C-terminal tails of preformed MTs does not induce MT stabilization [14,15]**.** On the other hand, acetylation modifies inter protofilament interactions by modulating K40 loop such that it increases stiffness and prevent damage from mechanical stress [16,17]. Cryo EM studies in combination with molecular dynamic (MD) simulations indicate that acetylation restricts the K40 loop motions and in deacetylated state, this loop undergo series of motions thereby increasing accessibility of enzyme α TAT1 which is known to be major acetyltransferase [18–20]**.** Our deacetylation/acetylation studies using rat and human sperm show that HDAC6 specific inhibitor—Tubastatin A (T) inhibited sperm motility and increased acetylated tubulin in rat sperm. Likewise, we also observed reduced acetylated tubulin in asthenozoospermic men [21,22]. These studies prompted us to investigate axonemal stability and whether impairment in stability has any influence on sperm motility.

Whether the key enzymes α TAT1 and HDAC6 and their corresponding PTMs acetylation/deacetylation regulate microtubule stability remains contentious. Experimental strategies wherein α TAT1 was either catalytically inactivated or knocked down using shRNA, revealed ambiguous observations with respect to microtubule stability [19,23]. In contrast, studies on HDAC6 indicated that abrogation of HDAC6 deacetylase activity either by site-directed mutagenesis or by its specific inhibitor, increases stability of microtubules in cells and speculated the function of HDAC6 as a putative MAP [24–26]. None of these studies confirmed the role of HDAC6 as a MAP.

More than a decade after discovery of α TAT1 and HDAC6, there is still dearth in literature pertaining to mechanistic role of these enzymes and their corresponding PTMs and other MT associated components in sperm flagella and motility. In this report, we have investigated stability of sperm axonemal MTs through isolation of polymerized axoneme from rat and human sperm. To consolidate our results regarding stability of sperm MT axoneme, we checked the expression of microtubule stabilizing proteins in normozoosperm and asthenozoosperm. Additionally, acetylated tubulin which is a hallmark of stability was also checked in these sperm. Available literature speculates role of HDAC6 as a MAP. In order to discern this in spermatozoa flagellar MTs, we used rat spermatozoa/sperm axonemes and determined HDAC6 binding and activity in the presence of its specific inhibitor. HDAC6 expression and activity was also determined in human sperm. Finally, *in vitro* polymerization and MT pellet down assays confirmed that HDAC6 binds efficiently to MT and inhibition of HDAC6 activity with TBSA probably causes a conformational change in HDAC6 thereby preventing its dissociation from MT, thus maintaining it in the polymerized state.

## Materials and methods

### Chemicals and reagents

Swine anti-rabbit IgG, Rabbit anti-mouse IgG, Goat anti rabbit IgG antibody (Dako, Glostrup, CPH, Denmark), Ac α-tubulin antibody, α-tubulin antibody, HDAC6 antibody, SAXO1 antibody (Sigma-Aldrich, St. Louis, MO, U.S.A), MDP3 antibody (outsourced from Genescript, Piscataway, NJ, U.S.A.), 2D Quant Kit (GE healthcare, Marlborough, MA, U.S.A.), ECL plus Western blotting detection reagent (GE healthcare, Little Chalfont, Buckinghamshire, U.K.); Western Blot Chemiluminescence HRP substrate (TAKARA BIO INC, Otsu, Shiga, Japan); Supersignal West Femto (Thermo Scientific, Rockford, IL, U.S.A); Westar Supernova chemiluminescence substrate (Cyanagen, Bologna, Italy); Tubulin Polymerization Assay Kit, Microtubule Binding protein Spin Down Assay Kit (Cytoskeleton Inc, Denver, CO, U.S.A.), HDAC6 Fluorimetric Drug Discovery Kit (Enzo Life Sciences Inc, Farmingdale, NY, U.S.A.), Reagents - Dimethyl Sulfoxide, Nocodazole, Tubastatin A (Sigma- Aldrich, St. Louis, MO, U.S.A.).

### Study system and ethics approval

Adult rat and human sperm were used for the study.

Adult male Holtzman rats aged 2.5–3 months were used. Rats were housed in four per cage under conditions of 14-h light and 10-h dark. Food and water were provided *ad libitum*. All animal care practices and experimental procedures pertaining to animal work carried out at the ICMR-National Institute for Research in Reproductive Health (ICMR-NIRRH), complied with the Committee for the Purpose of Control and Supervision of Experiments on Animals (CPCSEA) guidelines and were approved by the Institutional Animal Ethics Committee (IAEC) of ICMR-NIRRH (Project no. 13/17).

For human sperm, semen samples from 22 normozoospermic and 24 asthenozoospermic infertile individuals were collected after 3 days of abstinence. After liquefaction of the semen for 30 min to 1 h, the samples were evaluated according to World Health Organization Guidelines, 2010 [27]. Patients with a history of chronic infection, or those on medication, were excluded from the study. The semen samples consisting of more than 1 × 10^6^ pus cells were excluded from the present study. Samples that were hyper viscous and necrozoospermic were also excluded from the study. The sperm parameters for the recruited samples are described (Supplementary Table).

These individuals were recruited from the Infertility Clinic at ICMR-NIRRH, Mumbai, and ethics clearance was obtained from the Institute Clinical Ethics Committee (ICEC) of ICMR-NIRRH (Project no. 262/2014). The research has been carried out in accordance with the World Medical Association Declaration of Helsinki, and all subjects provided written informed consent.

### Isolation of sperm

For retrieving rat sperm, the animals were euthanised by CO_2_ asphyxiation, the epididymides adjoining the testes were dissected out and the caudal region of epididymides was excised and sperm were isolated from the caudal region by making incisions and allowing the release of sperm by incubating the teased tissue in Advanced Dulbecco’s modified Eagle’s medium (DMEM; Invitrogen, Carlsbad, CA, U.S.A.) at 34°C for 10–15 min to release the sperm. The supernatants were collected, centrifuged at 800*g* for 20 min and the sperm pellets resuspended to obtain sperm concentrations of 5 × 10^6^/ml. For experiments with the depolymerising agent Nocodazole (N) and HDAC6 inhibitor Tubastatin A (T), 5 × 10^6^ sperm in 5% CO_2_-equilibrated DMEM were incubated with 0.06% dimethyl Sulfoxide (DMSO; vehicle control), 50 μM N, 5 μM T or both at 37°C for 3 h. Sperm viability and motility were analyzed prior to and at the end of the incubation period. Sperm viability was evaluated using 0.5% eosin Y in 0.154 M NaCl. Equal volumes of eosin solution and sperm suspension were mixed and observed immediately under a microscope. Sperm heads stained dark pink were accounted as dead. Sperm motility was assessed by Computer Assisted Sperm Analysis (CASA; Hamilton Thorne Inc, Beverly, MA, U.S.A). For extraction of axoneme, the experiment was done with quadruplicates for each group. The sperm cells from the respective groups were pooled after 3 h and centrifuged in 0.1 M PBS at 800*g* for 20 min at 4°C. The sperm pellets thus obtained were used for further assays. For human sperm isolation, the semen samples on routine analysis were washed thrice in 0.1M PBS, pH 7.4, by centrifugation and pelleted at 800*g* for 30 min at 4°C and stored at −80°C, until further use. Depending on the total sperm count in individual samples, the number of spermatozoa to be used for each assay was determined.

### Sperm lysate preparation

Rat sperm: Sperm pellets obtained as described above were processed either for axoneme extraction or lysis in 15 mM TRIS-HCl buffer, pH 7.4, containing 0.34 M sucrose, 60 mM KCl, 15 mM NaCl, 0.65 mM spermidine, 2 mM EDTA, 0.5 mM EGTA, 0.05% Triton X-100, 1 mM DTT and 0.5 mM phenyl methyl sulfonyl-fluoride (HDAC6 lysis buffer) [28] followed by enzyme assays. For axoneme extraction, rat sperm pellets were subjected to flagella isolation following a published protocol [29]. Briefly, sperm pellets were sonicated at 65% output for 2 min with interval of 30 s. The sonicated sample was then layered on a sucrose gradient made using 7 ml each of 65, 70, and 75% sucrose in 0.1 M PBS, followed by centrifugation at 1,00,000*g* for 1 h at 4°C. The sperm flagella fraction was collected from the interface of 65 and 70% sucrose. The percent purity of the fraction was calculated by counting number of head in flagella fractions and flagella fractions with purity greater than 90% was used for further assays. For other experiments, sperm pellets were suspended in HDAC6 lysis buffer followed by homogenization using Fast prep -24 homogenizer (MP Biomedicals, Irvine, CA, U.S.A). The homogenized suspensions were incubated on ice at 4°C and then centrifuged to collect supernatant. Total protein content of the supernatants were determined using the 2-D Quant Kit.

Human sperm: Sperm pellets from the normal spermatozoa and asthenozoospermic groups were washed twice in 0.1 M PBS, pH 7.4, suspended in 2D- lysis buffer [22] comprises 7 M urea, 2 M thiourea, 4% CHAPS, 30 mM Tris, protease inhibitor cocktail (Roche Diagnostics, Indianapolis, IN, U.S.A.) and phosphatase inhibitor cocktail (Sigma Aldrich, St. Louis, MO, U.S.A.) and further homogenized and processed as described for rat sperm. The supernatant was quantified for total protein content using the 2D Quant Kit.

### Axoneme extraction

Rat sperm flagellar pellets and human sperm pellets were processed to enrich their axoneme fraction by solubilizing the sperm membrane with Triton X-100 as per the protocol described [30]. Equal number of sperm cells were used for axoneme extractions 35 × 10^6^ each for normozoosperm and asthenozoosperm, and 20 × 10^6^ per group for rat sperm. Briefly, following Triton X-100 treatment, rat sperm flagella or human sperm pellet were centrifuged at 1500*g* for 10 min, and the supernatants were collected and further subjected to ultracentrifugation at 47,800*g* for 20 min to pellet out the axonemes. The axonemal fractions were reconstituted in a buffer containing 87 mM PIPES, 36 mM MES, 1.4 mM MgCl_2_, 1 mM EGTA, pH 6.8 (PME buffer). The axonemal protein lysates were resolved on 10% SDS-polyacrylamide gels using the standard protocol [31] and transblotted to nitrocellulose (NC) membranes. As equal cell numbers were used for each group, equal volumes of axonemal lysates were loaded from each group. The blots were probed with antibodies to α-tubulin to determine the status of polymerized axoneme.

### HDAC6 enzyme assay

HDAC6 enzyme assay was performed using the Fluor-de- Lys HDAC6 fluorimetric drug discovery kit. This kit was used to determine the deacetylase activity in rat- and human sperm lysates as well as in axonemal lysates. Briefly, 25 μg of sperm lysates and sperm axonemal lysates used as a source of HDAC6 were incubated with 10 μM Fluor de Lys SIRT1 substrate in the absence or presence of 5 μM T, 50 μM N or both at 37°C for 2.5 h. Deacetylase activity was also determined in axonemal lysates of sperm treated with the drugs in which case 25 μg of lysates of the treated sperm were incubated with 10 μM of only the Fluor-de-Lys SIRT1 substrate at 37°C for 2.5 h. At the end of incubation, 50 μl 1× Fluor-de-Lys developer was added and the fluorescence measured at excitation and emission wavelengths of 360 and 460 nm, respectively. Fluor-de-Lys deacetylated standard and Fluor-de-Lys substrate incubated with human recombinant HDAC6 (recHDAC6) were always included in the assays as positive controls. The experiment was performed minimum three times with technical replicates in case of rat sperm and six different sperm samples in case of human sperm.

### *In vitro* polymerization assay

*In vitro* tubulin polymerization assays were performed using the Tubulin Polymerization Assay Kit containing >99% pure tubulin, of porcine origin. The microtiter plate was pre warmed to 37°C for 30 min prior to the assay. About 30 μM tubulin was aliquoted into the prewarmed wells without or with 10 μM N, 5 μM T or both along with rec HDAC6 in various combinations. The absorbance was recorded every min in the kinetic mode at 37°C for 1 h. The polymerization curve was plotted for all the reaction. The experiment was performed thrice.

### MT binding protein spin down assay

This assay is based on the principle that intact MT pellet down on centrifugation at 1,00,000*g*. Consequently, any protein associated with MT will co-pellet with MTs during centrifugation. This assay was performed with rec HDAC6 and purified tubulin using the MT binding protein spin down assay kit.

The tubulin protein in the kit was allowed to polymerize into MTs by incubating at 37°C for 20 min in 80 mM PIPES buffer, pH 7.0, containing 1 mM MgCl_2_, 1 mM EGTA, and 60% Glycerol (Cushion buffer). In order to stabilize the newly formed MTs, 2 mM Taxol was added. Following this, 6.15 μM recHDAC6 was added either singly or in combination with 5 μM T and further incubated at RT for 30 min. MAP fraction and Bovine serum albumin (BSA) provided with the kit were used as controls. Following incubation, the cushion buffer was placed at the bottom in the ultracentrifuge tube and carefully overlaid with the above reactions. These reactions were further subjected to ultracentrifugation at 1,00,000*g* for 30 min to pellet MTs. The supernatant and pellet fractions were separated to equal volumes of supernatant and pellet fractions. 2× sample loading buffer was added and these reactions were electrophoresed on 10% SDS-PAGE and then transblotted to NC membranes and the gels were also Silver stained.

### SDS-PAGE and Western blot analysis

The protein lysates were electrophoresed in varied amounts depending on the antigen of interest, on 12% SDS-PAGE at 100 V for 2.5 h and trans-blotted on NC membranes at 100 V for 1 h. The blots were then processed for Western analysis of α-tubulin, acetyl α-tubulin, SAXO1, MDP3 and HDAC6. Briefly, nonspecific binding was blocked by incubating the blots with 5% (wt/vol) non-fat dry milk (NFDM) in 0.1 M PBS for 1 h. For investigating the respective proteins, the transblotted NC membranes were probed with antibodies to α-tubulin (1: 40,000; monoclonal) to check polymerization in rat-and human sperm, and antibodies to acetyl α-tubulin (1:10,000; polyclonal), HDAC6 (1:100, monoclonal), SAXO1 (1:200, Polyclonal) and MDP3 (1:500, Polyclonal) to check expression of these proteins in human sperm. The blots were incubated with the respective antibodies overnight at 4°C or at RT for 2 h, followed by three washes with 0.1 M PBS containing 0.1% Tween 20 (PBST), and incubation with the appropriate secondary antibody (Rabbit anti-mouse for α-tubulin, acetyl α-tubulin and HDAC6; Swine anti-rabbit for SAXO1; and, Goat anti-rabbit for MDP3). All secondary antibodies were used at a dilution of 1: 3000 for 1 h at RT. The diluents used for primary and secondary antibodies were 1% NFDM in 0.1 M PBS for α-tubulin, acetyl α-tubulin and SAXO1, and 1% NFDM in 0.1% PBST for HDAC6 and MDP3. The unbound antibody was washed off with 0.1 M PBS containing 0.1% Tween 20 and the protein bands on the membrane were detected using chemiluminescence detection kit. The band volume intensities for each protein and the ponceau stained profiles of their respective lanes were quantified by densitometry on a gel documentation system using Gene Tools Version 3.6.4.1 (Syngene, Cambridge, U.K.), and the data were presented as intensity ratios (band intensity: ponceau lane intensity) and these were compared between the normal and asthenozoosperm. For axonemal lysates, equal volumes were loaded on SDS PAGE gels, as axonemal extraction was done from uniform number of sperm cells for all the groups.

### Statistical analysis

All data were analyzed for statistical significance between two groups by unpaired Student’s t-test and between multiple groups by One-way ANOVA with Bonferroni post-test correction using GraphPad Prism version 8.4.0 (GraphPad Software Inc., San Diego, CA, USA). All experiments were carried out at least thrice unless stated otherwise.

## Results

### The status of polymerized axoneme in sperm

**Figure 1:**
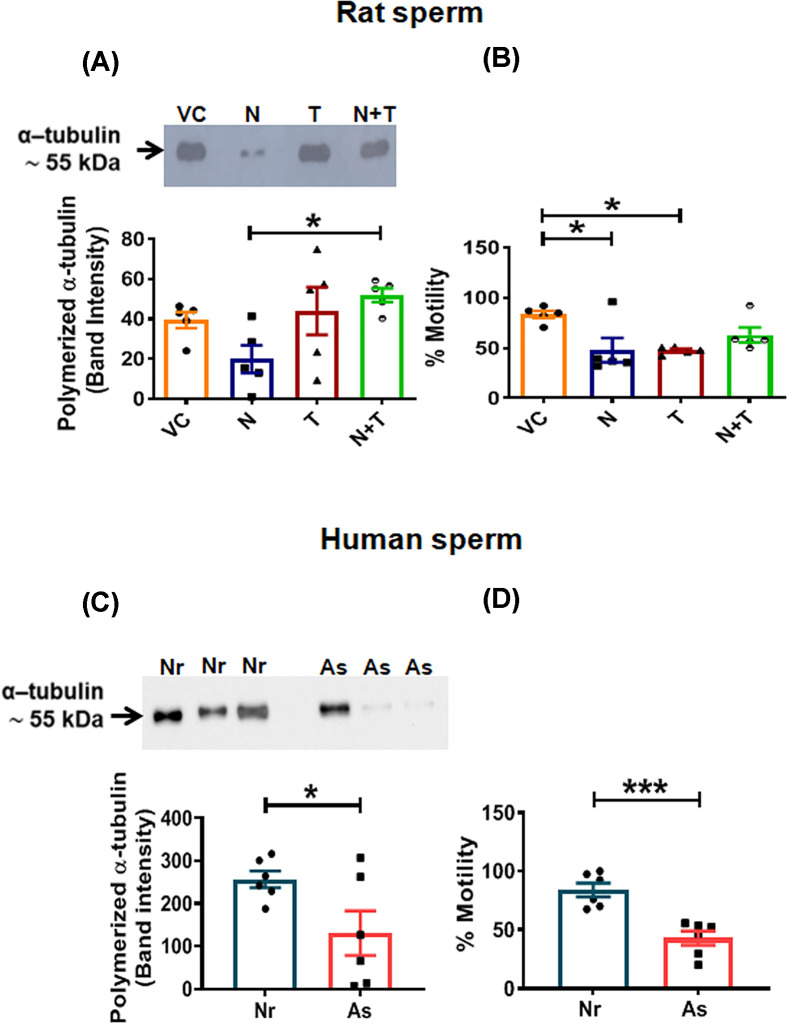
Polymerized axoneme (α-tubulin) in flagella of rat and human sperm (**A**) Representative Western blot of α-tubulin (polymerized axoneme) in axonemal lysates of rat caudal sperm treated without (VC) or with Nocodazole (N), Tubastatin (T) and both for 3 h at 37°C; followed by axoneme extraction and detection of α-tubulin in the axonemal lysate; Corresponding graph shows densitometric analysis of the same. (**B**) Graph represents percent motility corrected for viability after 3 h of incubation of rat caudal sperm with the drugs. (**C**) Representative Western blot of α-tubulin (polymerized axoneme) in axonemal lysates of Normoozoosperm (Nr) and Asthenozoosperm (As). (**D**) Graph represents values for sperm motility of respective semen samples corrected for viability. Data represents mean ± S.E.M. from five independent experiments with 20 × 10^6^ sperm/grp/experiment for rat sperm, and six samples (*n* = 6) with 35 × 10^6^ sperm/sample in case of human sperm; *P ≤ 0.05; ***P ≤ 0.001; VC, vehicle control.

The axonemal lysates obtained from equal number of sperm treated with either depolymerizing agent Nocodazole (N), HDAC6 inhibitor Tubastatin A (T) or both, were electrophoresed by SDS-PAGE followed by Western blot analysis. The status of polymerized axoneme was determined by densitometric analysis of α-tubulin expression in the four groups (Figure 1A). Expression of α-tubulin was used as a surrogate indicator for polymerized axoneme. Though statistically insignificant, N alone showed modest reduction in α-tubulin compared with that in vehicle control (*P* = 0.48) and T (*P* = 0.99). Expression of α-tubulin was significantly increased in rat sperm treated with the combination of N and T as compared with N alone (*P* = 0.04). Sperm viability that was 85 – 90% before treatment with the drugs dropped to 65 –80% at the end of the incubations. Percent viability was 78 ± 0.63 for VC, 75.4 ± 1.04 for N, 73.4 ± 0.73 for T and 69.6±1.61 for the N+T treated sperm (Supplementary Figure S1). Hence, the motility values for sperm from each group were normalized to their respective viability values. Sperm motility was observed to be significantly reduced with N (*P* = 0.02) as well as with T (*P* = 0.02) but not with the combination (Figure 1B). The axonemes from intact sperm of normozoospermic and asthenozoospermic individuals were similarly analyzed. The axonemal α-tubulin was significantly lower in asthenozoosperm as compared with normozoosperm (*P* = 0.046) (Figure 1C). Sperm motility of individuals from both the groups is shown in Figure 1D.

### Deacetylase activity in sperm

The presence of deacetylase activity of HDAC6 in rat caudal sperm was determined indirectly by using HDAC6 inhibitor Tubastatin A [21]. Activity was determined in rat sperm lysate, or sperm axonemal lysate treated with the drugs and in axonemal lysates of sperm treated with the drugs.

**Figure 2:**
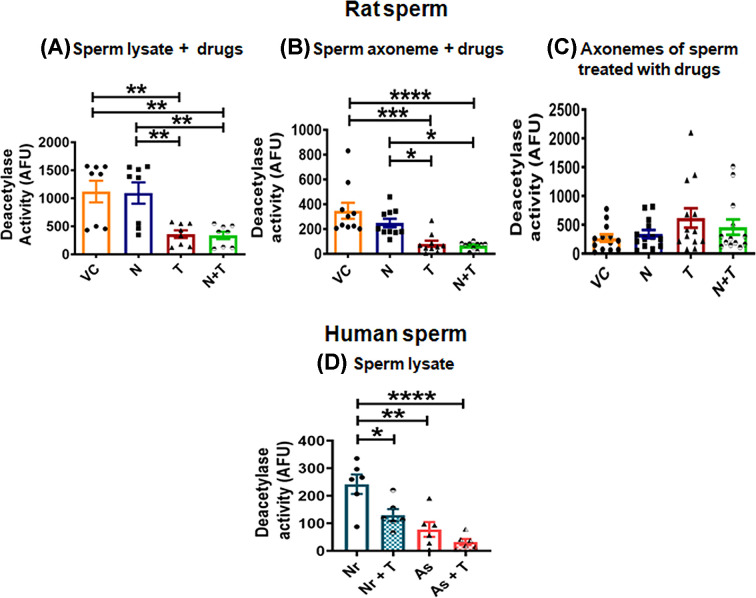
Deacetylase activity in rat and human sperm Deacetylase activity in (**A**) sperm lysates incubated with Nocodazole (N; 50 μM), Tubastatin A (T; 5 μM) or both (N + T, 50 and 5 μM), (**B**) sperm axonemes incubated with N, T or both N + T. (**C**) Deacetylase activity in axonemes of rat sperm treated with drugs. Deacetylase activity assay was performed using HDAC6 Fluorimetric Drug Discovery Kit. (**D**) Deacetylase activity in sperm lysates of normozoosperm and asthenozoosperm. Statistical significance was determined by one-way ANOVA (A** –** C) and unpaired t-test (D) with significance level set at *P ≤ 0.05, **P ≤ 0.01, ***P ≤ 0.001, ****P ≤ 0.0001. Data represents mean ± S.E.M. of arbitrary fluorescence units (AFU) from minimum three biological replicates with technical triplicates for each in case of rat sperm and six samples (*n* = 6 per group) in case of human sperm. Deacetylase activity is significantly reduced in rat sperm lysates and sperm axonemes exposed to T and N + T but not in axoneme lysates of sperm treated with T and N + T. In humans, deacetylase activity is significantly reduced in asthenozoosperm as compared with normozoosperm. Its reduction on sperm exposure to HDAC6 specific inhibitor Tubastatin A, in both the group of men, indicates that HDAC6 activity is reduced.

The activity was significantly reduced in sperm lysates treated with either T (*P* = 0.005) or N + T (*P* = 0.004) as compared with that observed in VC and N treated sperm lysates. Activity in N (*P* = 0.999) was comparable to that seen in VC (Figure 2A). Interestingly, deacetylase activity was found to be remarkably reduced even with sperm axonemal lysates incubated with either T (*P* ≤ 0.001) or N+T (*P* ≤ 0.0001) as compared with VC. Similar observations were seen in comparison to N with *P* values for T and N+T being *P* ≤ 0.05 and *P* ≤ 0.05, respectively. Deacetylase activity in N did not show a significant change from that seen in VC (Figure 2B). However, no noteworthy differences were seen in the deacetylase activity of axonemal lysates when intact sperm were treated either with N, T or N + T (Figure 2C). Deacetylase activity was also determined in human sperm lysates incubated without or with T. Deacetylase activity was significantly lower in asthenozoosperm (*P* = 0.0011) as compared with normozoosperm (Figure 2D). In the presence of HDAC6 specific inhibitor T, the deacetylase activity was significantly reduced in normozoosperm (*P* = 0.03) and moderately reduced in asthenozoosperm (*P* = 0.99) compared with their respective activities in the absence of T (Figure 2D). On comparing the relative reduction post treatment between the two groups, it was insignificant (Supplementary Figure S2).

### Expression of HDAC6 in sperm axonemal lysates

**Figure 3:**
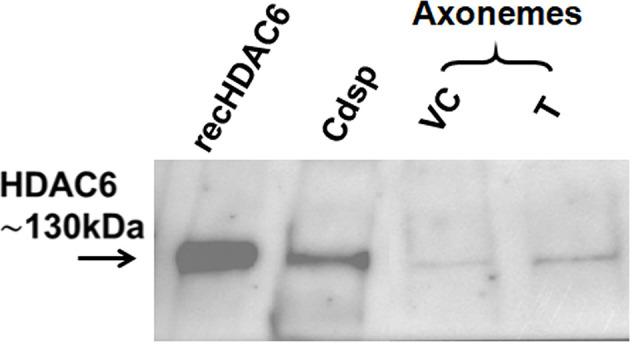
Enhanced expression of HDAC6 in sperm axonemal lysates in presence of Tubastatin A Representative Western blot shows a band of HDAC6 at ∼130 kDa in axonemal lysates of sperm treated without or with T; VC: Vehicle control; T: Tubastatin A; Recombinant HDAC6 (recHDAC6) and rat caudal sperm lysate (Cdsp) were used as positive controls for HDAC6 expression. This experiment was done twice. In order to be able to detect HDAC6 expression on axoneme, 16 × 10^7^ sperm each for the ‘VC’ and ‘T’ group were used per experiment.

Axonemal lysates of rat sperm treated without or with T were analyzed for expression of HDAC6 by Western blot analysis. Rec HDAC6 and caudal epididymal sperm lysates used as positive controls showed a band for HDAC6 at ∼130 kDa. While expression of HDAC6 was low in axonemal lysate of untreated sperm, a relatively intense band was seen in axonemal lysates of sperm treated with T (Figure 3).

### HDAC6 and MT polymerization

**Figure 4:**
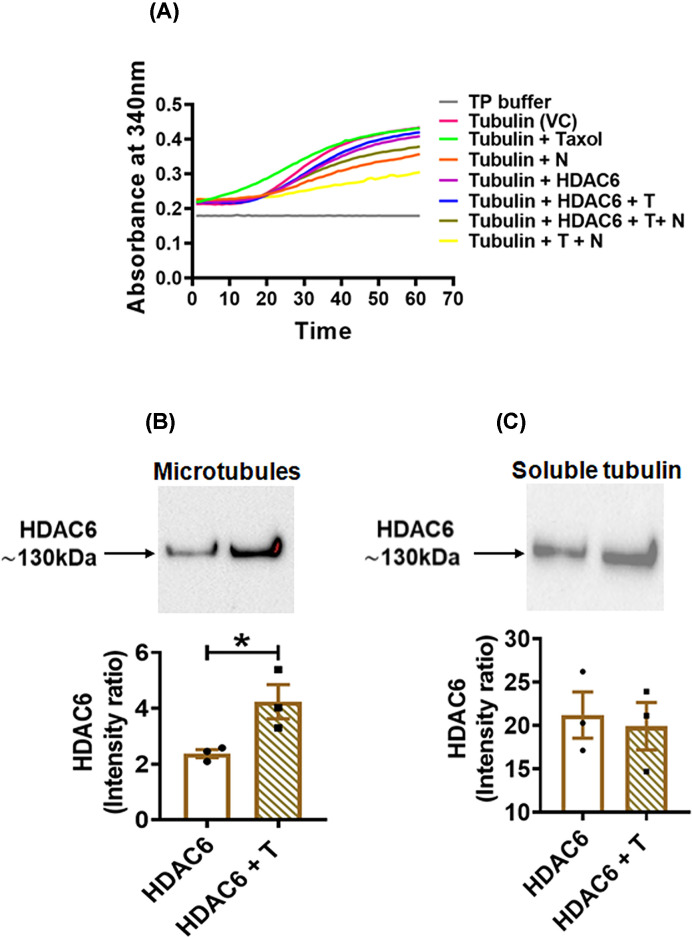
*In vitro* tubulin polymerization and detection of HDAC6 in Microtubule pellet and supernatant fractions (**A**) *In vitro* Microtubule polymerization assay was performed wherein soluble tubulin was incubated with microtubule stabilizing agent (Taxol; 10 μM), or Microtubule depolymerizing agent Nocodazole (N; 10 μM). The influence of HDAC6 on tubulin polymerization was determined by incubating tubulin with recombinant HDAC6 (HDAC6) alone or along with Tubastatin A (T; 5 μM) or 10 μM N, or both. Absorbance was recorded every min in the kinetic mode at 37°C for 1 h. The polymerization curve was plotted for each reaction. Microtubule spin down pellet assay was carried out to analyze HDAC6 binding to microtubules and soluble tubulin in order to ascertain its putative role as microtubule-associated protein (MAP). Preformed microtubules were incubated along with recombinant HDAC6 either without or with T, at RT for 30 min followed by centrifugal separation of pellet containing intact microtubules (**B**) and supernatant containing soluble tubulin (**C**). Data represents mean ± S.E.M. from values of minimum three independent experiments; *P ≤ 0.05.

The influence of HDAC6 on MT polymerization was also studied *in vitro* using an *in vitro* polymerization assay performed with purified tubulin and rec HDAC6 in combination with T and N+T. Tubulin alone or in combination with Taxol that were used as positive controls showed gradual increase in absorbance with time. Tubulin in presence of HDAC6 alone or in combination with T (HDAC6 + T) showed gradual increase in absorbance that was almost comparable to that seen in the positive controls. In the presence of N, the absorbance did not increase as much and was lower than that seen with the positive controls, HDAC6 and HDAC6 + T. The absorbance when tubulin was incubated with all three components together (T + N + HDAC6), also increased with time, although lower than with HDAC6, or HDAC6 + T, the increase was certainly higher compared with tubulin incubated with N alone. Tubulin incubated with T + N showed only slight increase in absorbance over time. (Figure 4A). The MAP like characteristic of HDAC6 was explored by performing Microtubule spin down pellet assay *in vitro*. The presence of HDAC6 was observed in the pellet (MT) as well as supernatant (soluble tubulin) fractions. The presence of HDAC6 was significantly higher (*P* = 0.04) in the MT fraction when MTs were incubated with HDAC6 and T as compared to that when incubated with only HDAC6 (Figure 4 B,C). The presence of HDAC6 in the soluble tubulin fractions were comparable.

### Expression of acetyl α-tubulin, HDAC6, and microtubule stabilizing proteins in human sperm

**Figure 5:**
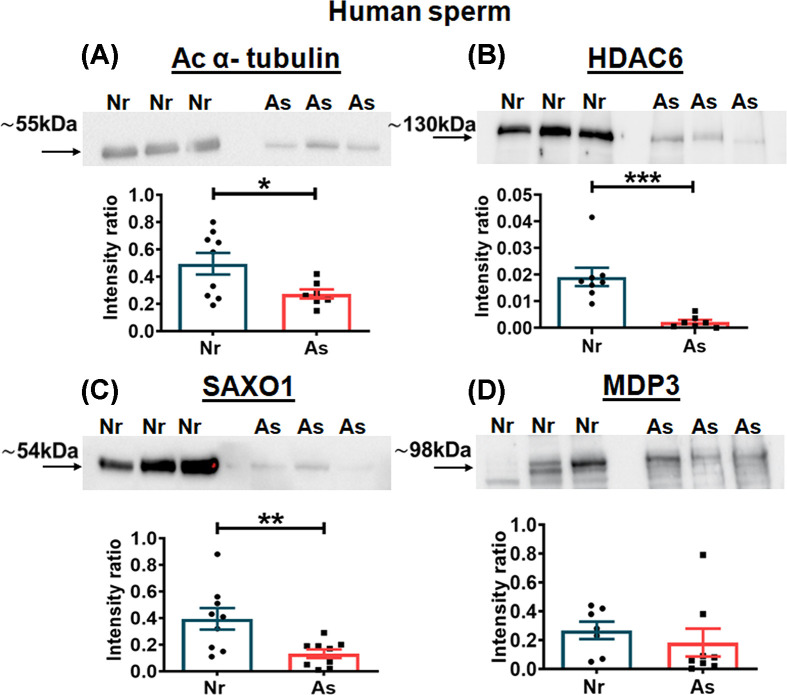
Expression of acetyl α-tubulin, HDAC6, and microtubule stabilizing proteins in human sperm Representative blots and densitometric analysis of (**A**) acetyl α-tubulin (Ac α-tubulin), (**B**) histone deacetylase 6 (HDAC6), (**C**) stabilizer of axonemal protein 1 (SAXO1) and (**D**) MAP 7 domain containing protein 3 (MDP3) in Normozoosperm (Nr) and Asthenozoosperm (As); n = minimum 6 per group. Statistical significance was determined using unpaired t-test. *,**, and *** denote significance at *P* ≤ 0.05, *P* ≤ 0.01, and *P* ≤ 0.001, respectively. Values are mean ± S.E.M.

The status of acetyl α-tubulin, HDAC6, and microtubule stabilizing proteins namely stabilizer of axonemal protein 1 (SAXO1) and MAP7 domain containing protein 3 (MDP3) was investigated in human sperm lysates by Western blot analysis. A band at ∼55 and ∼130 kDa was seen for acetyl α-tubulin and HDAC6, respectively. Acetyl α-tubulin was significantly reduced in asthenozoosperm (*P* = 0.024) as compared with Normozoosperm (Figure 5A). HDAC6 was also reduced significantly in Asthenozoosperm (*P* = 0.0001) as compared with normal sperm (Figure 5B). The band intensities for SAXO1 were significantly reduced in asthenozoosperm (*P* = 0.008) as compared with normozoosperm. However no significant difference was seen for MDP3 (*P* = 0.487). The band at ∼54 and ∼98 kDa was observed for SAXO1 and MDP3, respectively (Figure 5C,D).

## Discussion

Microtubule (MT) dynamics or stability of microtubules has been extensively studied and discussed in somatic cells and lower ciliated/flagellar organisms in order to comprehend the mechanism underlying cancer, nervous disorders and ciliopathies since the MTs are highly active in regulating various inter- and intracellular functions [2]. However, mature sperm flagella are composed of highly stable protofilaments and other accessory structures required for motility. The stability of MTs can be attributed to post-translational modifications and MT associated proteins. It has been reported earlier that acetylation accumulates on soluble pool of tubulin [32,33] and on exposure to MT stabilizing drug Taxol, acetylation remarkably increases and might be consequence of stabilization of the tubulin polymer [34]. Acetylation at K-40 residue of α-tubulin is catalyzed by αTAT1, which is conserved in ciliated organisms [35], whereas its deacetylation is catalyzed by HDAC6 [36,37] and Sirtuin 2 [38]. Studies *in vitro* and in various cell lines suggest that αTAT1 and HDAC6 may have a regulatory role in maintaining dynamicity of MTs. Studies on knockout and overexpression of αTAT1 [19,23], and HDAC6 indicated a role for acetylation/deacetylation in MT stability [37]. Interestingly, several reports indicate that the inhibition of deacetylase activity of HDAC6 and not its depletion is responsible for MT stability [24–26]. Our studies in rat model have shown the presence of HDAC6 in sperm and that inhibition of HDAC6 deacetylase activity adversely affects sperm motility [21]. Our studies using human sperm, revealed reduced acetylation of α-tubulin in asthenozoosperm [22]. These observations prompted us to explore MT dynamicity in sperm and its implications to sperm motility.

In order to assess the role of HDAC6 in stability of MTs in sperm, we used rat- as well as human sperm. Our results demonstrate that HDAC6 binding to its specific inhibitor Tubastatin A protects sperm axonemal MTs from the depolymerizing effect of nocodazole as reflected by the increase in polymerized axoneme when sperm are treated with N+T together as compared with nocodazole alone. This binding also appears to obliterate the negative effect of T on sperm motility (Figure 1A,B). This observation further substantiates our earlier reported observation of HDAC6 having a role in sperm motility [21]. These observations also suggested that on inhibition of HDAC6, it remains bound to the axoneme and this possibly prevents MT depolymerization. These interesting observations encouraged us to explore the status of polymerized axoneme in human sperm. The polymerized axoneme was significantly reduced in individuals with poor sperm motility as against that seen in normozoospermic men (Figure 1C,D). This may either be due to possible differences in the lengths of the flagella between the two groups of sperm or it may be suggestive of a relatively rigid and stabilized axoneme in sperm with normal motility.

Having observed increase in polymerized axoneme in sperm treated with N+T, as compared with N, we further studied deacetylase activity in rat sperm under three conditions and using the Fluor-de- Lys HDAC6 Fluorimetric Kit [21]. As expected deacetylase activity was significantly reduced when rat caudal sperm lysates were treated with either Tubastatin A alone or in combination with Nocodazole. But what intrigued us was that similar observations were seen when sperm axonemal lysates were treated with the drugs (Figure 2A,B). This further strengthened our observations that HDAC6 may indeed be present on the sperm axoneme and may likely act as a MAP. However when sperm were first treated with the drugs and then their axonemes were extracted and deacetylase activity of the axonemal lysate was examined, although deacetylase activity was seen in all groups, no differences were observed between any of the groups. This is perplexing because under these conditions differences were noted in polymerized tubulin in N+T group compared with N as also in motility (Figure 1A,B). Additionally, even HDAC6 expression on axoneme could be seen and in fact it was enhanced in the axonemal lysates of sperm treated with Tubastatin A thus endorsing the presence of HDAC6 on the sperm axoneme and strengthening our contention for its role as a MAP (Figure 3). Deacetylase activity in asthenozoosperm lysates was remarkably reduced in individuals as compared with normozoosperm. Although we measured the total deacetylase activity, its equal inhibition by Tubastatin A in both the groups indicate that most of the deacetylase activity came from HDAC6 (Figure 2D and Supplementary Figure S2).

The *in vitro* polymerization assay and microtubule-binding protein spin down assay further substantiate the role of HDAC6 in polymerization of microtubules (Figure 4A,B). That inhibition of HDAC6 prevents its dissociation from MT has been reported earlier in B16F1 and MCF7 cell lines [25,26]. Ours is the first report demonstrating it in the germ cell namely sperm. *In vitro* polymerization assays revealed the role of HDAC6 in MT polymerization as in the presence of HDAC6, without or with Tubastatin A, enhanced tubulin polymerization was seen as compared with that seen with tubulin incubated with nocodazole alone. Additionally, tubulin incubated with all three components together (Tubulin + T + N + HDAC6), although showed polymerization lower than that seen with HDAC6, or HDAC6 + T, the increase was certainly higher as compared to tubulin incubated with Nor N + T (Figure 4A). Significantly increased presence of HDAC6 was seen in the MT when tubulin was incubated with HDAC6 and Tubastatin A as compared with tubulin incubated with HDAC6 alone. The increased presence may be because Tubastatin on binding to HDAC6 associated with the MT prevents HDAC6 dissociation from the MT thereby preventing the depolymerization of the MT and thus increasing the MT stability. This has been reported previously in cell lines [25,26]. So as can be seen in Figure 4B, there is more of the intact MTs in the pellet fraction of ‘HDAC6 + T’ lane. This suggests that for polymerization to happen or to be maintained, HDAC6 must remain associated with tubulin (Figure 4B). The presence of HDAC6 was also seen in the fraction containing soluble tubulin which was not surprising as HDAC6 is known to bind to polymerized as well as soluble tubulin [39]. Latest report on HDAC6 binding to microtubules indicated that the N terminal part of HDAC6 called as microtubule-binding domain (MBD) was responsible for MT recognition as well as deacetylation. The group also reported that the deacetylase activity of HDAC6 is not required for its binding to MT [40]. Our observations and this reported evidence suggest that apart from functioning as a deacetylase, HDAC6 could also function as a MAP. In the case of sperm it may regulate sperm motility by maintaining the rigidity/flexibility of the sperm axoneme.

MAPs and microtubule inner luminal proteins (MIPs) decorate inner lumen as well as outer interface of microtubules to perform wide range of functions. The class of microtubule stabilizing proteins, MAP7 domain containing protein 3 (MDP3) and stabilizer of axonemal protein 1 (SAXO1), have been reported to regulate cilium axonemal length and promote microtubule assembly in Hela cells, and *Trypanosome Brucei* & RPE1 cells, respectively [41–43]. SAXO1 is also reported to be present across the length of human sperm flagellum where it colocalizes with α-tubulin [43]. Their contribution with respect to sperm axoneme and role in modulating sperm motility remained unexplored. We therefore investigated the expression of these proteins along with HDAC6 and acetyl α-tubulin in human sperm. The expression of SAXO1, HDAC6, and acetyl α-tubulin was significantly reduced in asthenozoosperm as compared with that seen in normozoosperm (Figure 5). As acetyl α-tubulin was reduced, it came as a surprise that the enzyme deacetylating it should also be low in asthenozoosperm. We expected an increase in HDAC6 activity and expression, and decrease in polymerized tubulin in asthenozoospermic men as previous studies from our laboratory showed reduced expression of acetyl tubulin in asthenozoospermic men [22]. In the present study too, we have observed lower expression of acetyl tubulin in asthenozoospermic men. But what perplexed us was that whilst we did see a decrease in polymerized tubulin in asthenozoospermic men, HDAC6 expression and deacetylase activity too were reduced. This is a novel observation and possibly suggests that tubulin acetylation–deacetylation is compromised in asthenozoosperm. This may likely be responsible for the lower deacetylase activity and reduced axonemal polymerization observed in sperm of asthenozoospermic men. Studies using 293T cells and HeLa cells show that MDP3 binds to and regulates HDAC6 on the MT and also controls MT stability [41,44]. Going by our observations of increased HDAC6 expression and decreased deacetylase activity in rat sperm incubated with Tubastatin A and also decreased HDAC6 activity in sperm of asthenozoospermic men, we expected MDP3 to be altered in asthenozoosperm, surprisingly MDP3 expression was unaffected in asthenozoosperm.

To summarize, we have shown that HDAC6 is a MAP and has a role in MT stability of sperm flagellar axoneme and that stability of axonemal microtubules is affected in sperm with poor motility. These observations taken together suggest that MAPs and the threshold levels and balance between MT acetylation/deacetylation are required in order to maintain MT dynamicity in sperm for effective sperm motility. Our ongoing efforts are now focused on identification of novel factors including MIPs, MAPs and HDAC6-interacting proteins that may probably contribute to mechanism/s underlying axoneme driven sperm motility.

## Supplementary Material

Supplementary Figures S1-S2 and Supplementary Table S1

## Data Availability

Raw data associated with the paper is available and can be accessed, by contacting the authors.

## Abbreviations

αTAT1: α-tubulin acetyl transferase 1
FS: fibrous sheath
HDAC6: histone deacetylase 6
IFT: intraflagellar transport
MAP: microtubule-associated protein
MDP3: MAP7 domain containing protein 3
MIP: microtubule inner luminal protein
ODF: outer dense fibre
SAXO1: stabilizer of axonemal protein 1
