# Supplementary material for: Acetylation/deacetylation and microtubule associated proteins influence flagellar axonemal stability and sperm motility
Source: Biosci Rep. 2020 Dec 2;40(12):BSR20202442. doi: 10.1042/BSR20202442 (PMC7711059; doi:10.1042/BSR20202442)
Supplement: Supplementary Figures S1-S2 and Supplementary Table S1 [file BSR-2020-2442_supp.pdf]

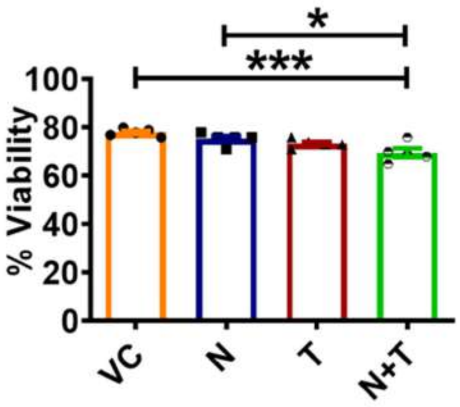

Supplementary figure 1. Viability analysis for rat sperm treated with Nocodazole (N) , Tubastatin A (T) or N+T.
Sperm viability was determined prior to- and after exposure to either N, T, or both, for 3h. The viability of rat sperm was determined using 0.5% eosin in 0.154M of NaCl. Data represents Mean ± SEM from minimum three independent experiments. *P < 0.05; ***P < 0.001

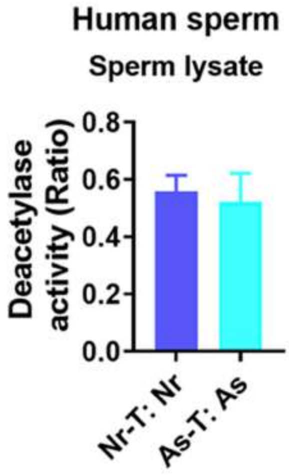

Supplementary figure 2: Deacetylase activity (ratio) in human sperm.
Deacetylase activity was examined in sperm lysates of normozoospermic- (Nr) and asthenozoospermic (As) men in the absence or presence of HDAC6 inhibitor Tubastatin A (T). Reduction in deacetylase activity on T exposure was observed in both the groups. To determine whether the extent of reduction differed between the two groups, we plotted the ratios of AFU values of treated normozoosperm lysates (Nr-T): untreated (Nr) and treated asthenozoosperm lysates (As-T): untreated (As). From the graph it is evident that the extent of reduction was similar in both the groups.

Supplementary Table

Semen parameters of Normozoospermic and Asthenozoospermic men.

Semen parameters	Values* (min - max)	
	**Normozoospermic men**	**Asthenozoospermic men**
**Semen volume (mL)**	2.28 ± 0.76 (0.8 – 4)	2 ± 0.71 (0.8 – 3.5)
**Sperm concentration (million /mL)**	77.70 ± 50.09 (21.65 - 273.75)	49.4 ± 23.97 [a] (15.6 - 119.38)
**Viability (%)**	77.32 ± 0.05 (69 - 88)	65.54 ± 0.06 [b] (59 – 80)
**Motility (%)**		
Progressive	55.45 ± 0.10 (36 - 73)	15.71 ± 0.07 [b] (0 – 28)
Non-progressive	11.27 ± 0.04 (6 – 23)	14.88 ± 0.06 [a] (0 – 29)
Immotile	31.55 ± 0.10 (16 – 49)	68.88 0.12 [b] (51 – 100)

* Values are Mean ± SD

[a] $p \leq 0.05$; [b] $p \leq 0.0001$
